# Accurate Guitar Tuning by Cochlear Implant Musicians

**DOI:** 10.1371/journal.pone.0092454

**Published:** 2014-03-20

**Authors:** Thomas Lu, Juan Huang, Fan-Gang Zeng

**Affiliations:** 1 Department of Otolaryngology – Head and Neck Surgery, University of California Irvine, Irvine, California, United States of America; 2 Mind-Brain Institute, Johns Hopkins University, Baltimore, Maryland, United States of America; University of Southern California, United States of America

## Abstract

Modern cochlear implant (CI) users understand speech but find difficulty in music appreciation due to poor pitch perception. Still, some deaf musicians continue to perform with their CI. Here we show unexpected results that CI musicians can reliably tune a guitar by CI alone and, under controlled conditions, match simultaneously presented tones to <0.5 Hz. One subject had normal contralateral hearing and produced more accurate tuning with CI than his normal ear. To understand these counterintuitive findings, we presented tones sequentially and found that tuning error was larger at ∼30 Hz for both subjects. A third subject, a non-musician CI user with normal contralateral hearing, showed similar trends in performance between CI and normal hearing ears but with less precision. This difference, along with electric analysis, showed that accurate tuning was achieved by listening to beats rather than discriminating pitch, effectively turning a spectral task into a temporal discrimination task.

## Introduction

Pitch is such a fundamental component of music that professional musicians can demonstrate an enhanced ability to detect smaller frequency differences compared to non-musicians [1]. In addition to general left and right hemisphere asymmetry between processing speech and music [2], musicians' brains can have further anatomical and functional changes due to their extensive musical training [3], [4], [5], [6], [7]. In contrast, due to both auditory deprivation and technological limitations, cochlear implant (CI) users generally perform poorly in music-related tasks [8], [9], [10], [11]. Whereas normal hearing (NH) listeners can detect a pitch difference of 1% or less, CI users on average require 10% or more [12]. For comparison, 6% or better is needed to hear a semitone difference in music, e.g., B versus B-flat.

In CI, the sensation of pitch is generated through both place and rate (periodicity) of electrical stimulation [13]. Place pitch depends on the tonotopic organization in the cochlea such that stimulations closer to the base produce increasingly higher perceived pitches [14]. Because modern devices have limited numbers of electrodes, 12–22, compared to nearly 3000 inner hair cells in NH listeners, place pitch is severely compromised in CI. The effective number and independence of these electrodes are further reduced by their broad and overlapping electric fields [15]. Furthermore neural degeneration in the cochlea from lack of inputs can distort pitch perception [16]. Consequently, it is no surprise that place coding of pitch is exceedingly poor in CI.

Alternatively, temporal pitch (or rate pitch) can be produced directly from the rate of stimulation pulses in the CI. In contrast to poor place coding, the microsecond timing of the stimulation pulses is theoretically precise enough to encode the entire audible range of frequencies. However, current speech processing strategies often discard fine timing information and use amplitude-modulated, high-rate pulse trains with fixed inter-pulse intervals [17], [18]. Even if the clinically used speech processing strategy is bypassed under laboratory conditions to accurately present rate pitch, pitch perception remains poor as CI users still have difficulty discriminating between pulses rates that are above 300 Hz [13]. This means that musical notes above middle C (261 Hz) would not be well distinguished when temporally coded by stimulation pulse rate alone. One possible explanation is that auditory deprivation leads to abnormal processing of sound even if hearing is partially restored by a cochlear implant [19]. In CI users, neural correlates for auditory processing of tones and musical sounds have been shown to be impaired [20], [21]. Furthermore, the continued delivery of spectrally impoverished information could limit the recovery towards normal auditory function. Given these factors, temporal pitch appears to be neither adequately encoded nor adequately perceived to be of use for music appreciation.

Despite this well-documented difficulty with CI pitch perception, there are musicians that continue to perform even after receiving a cochlear implant. Do these individuals have better pitch perception than typical CI users? In NH musicians, cortical plasticity can provide enhancement of auditory processing compared to non-musicians [3], [7], [22], [23]. Because the etiology and history of deafness of CI users vary widely, it is unclear to what extent they benefit from prior musical training and continued practice after CI implantation. Can a musically-trained individual with CI better extract pitch information compared to a CI user who was not musically trained?

For a CI user, tuning an instrument by ear via CI is a seemingly impossible task given the difficulty in perceiving pitch. Here we report not only the unexpected and accurate tuning of a guitar by a CI musician but also positive identification of the acoustic cue that is responsible for such accuracy. Furthermore, we provide direct comparisons between acoustic and electric pitch perception in two additional CI users who have normal, contralateral hearing. One was a musician, and the other was not. The results may help us understand whether a musically trained auditory system can better extract pitch from a device that offers only poor spectral information.

## Methods

### Subjects

Recruitment and experimental protocols for this study were approved by the University of California Irvine Institutional Review Board. Subjects provided written informed consent prior to the start of the experiments.

The first CI musician in this study, CI1, was a 35 year old male who suffered profound bilateral hearing loss in 2000. He was implanted unilaterally in the right ear in 2002 with a Nucleus 24 Freedom (Cochlear Ltd., Lane Cove, Australia). With 99% sentence recognition for HINT sentences in quiet, he was a star user. However, he could only recognize 55% of familiar melodies when rhythm cues were removed. The second subject, CINH001, was a 49 year old male and a professional musician (drums and guitar) and audio engineer. He lost hearing in his right ear in 2004, cause unknown, and was implanted with a HiRes 90K (Advanced Bionics, Valencia, CA) in 2005 to treat severe tinnitus. His non-implanted, left ear had normal hearing thresholds (<20 dB HL) except for a mild loss at 35 dB HL at 4000 Hz. With CI alone, he could recognize 70% sentences but only 7% familiar melodies without rhythm. The third subject, CINH002, was a 40 year old female but not a musician. In 2010, she suddenly lost hearing in her left ear and experienced the onset of severe and persistent tinnitus. She was implanted 6 months later with a PULSARci100 (MED-EL, Innsbruck, Austria) in an attempt to manage the tinnitus. Her normal, non-implanted right ear had normal thresholds (<10 dB HL). With CI alone, speech recognition was 45% and melody recognition was 8%. Both CINH001 and CINH002 had near perfect scores for speech and music when listening with their normal hearing ears.

### Psychophysical procedure

Pitch matching accuracy was assessed using custom software in MATLAB (Mathworks, Inc, Natick, MA) with an interface designed to approximate the tuning process of a musical instrument. Stimuli consisting of 1 s pure tones (5 ms on/off ramp) were generated at 44.1 kHz, output to the subject's speech processor using a direct connect cable, and presented at a comfortable loudness level. For CI1, the stimulus level was randomly roved by ±5 dB and ±10 dB to reduce loudness cues. Since CINH001 did not appear to take much advantage of loudness cues with his CI, level roving was skipped for this subject. Two tones were generated with randomized frequencies, both in the range of 100–400 Hz, 400–800 Hz, or 800–1600 Hz, with 3 or 4 repetitions in each range to get a broad representation of frequencies. The task was to manually adjust the pitch of the first tone to the second by pressing buttons on screen. Adjustment increments available were ±100 Hz, ±15 Hz, ±1 Hz and ±0.1 Hz. In the first condition, the two tones were presented simultaneously to imitate the way CI1 tunes the guitar. In the second condition, the tones were played sequentially with a 500 ms silent interval to eliminate beats and make pitch the primary cue. As control, the normal hearing ear of CINH001 was tested in a sound proof room using headphones (HDA 200, Sennheiser, Old Lyme, CT). The match error is defined as the absolute value of the frequency difference between the reference tone and the final frequency of the adjustable tone.

For CINH002, after initial trials with the above methods, the testing procedure was modified to reduce the difficulty by setting the starting frequency of the adjustable tone to be lower than the reference tone. This alteration can be justified because stringed instruments are typically loosened, lowering the pitch, before being brought up to tune. Tones were played via direct connect cable (to CI) or speakers (to NH). The 100–400 Hz range was not tested as her clinical processor had a 500 Hz low frequency cutoff for the most apical electrode. We tested 5 repetitions in for each of the 400–800 Hz and 800–1600 Hz range for both CI and NH ears. The trials using the CI had minimum frequency of 540 Hz.

## Results

**Figure 1:**
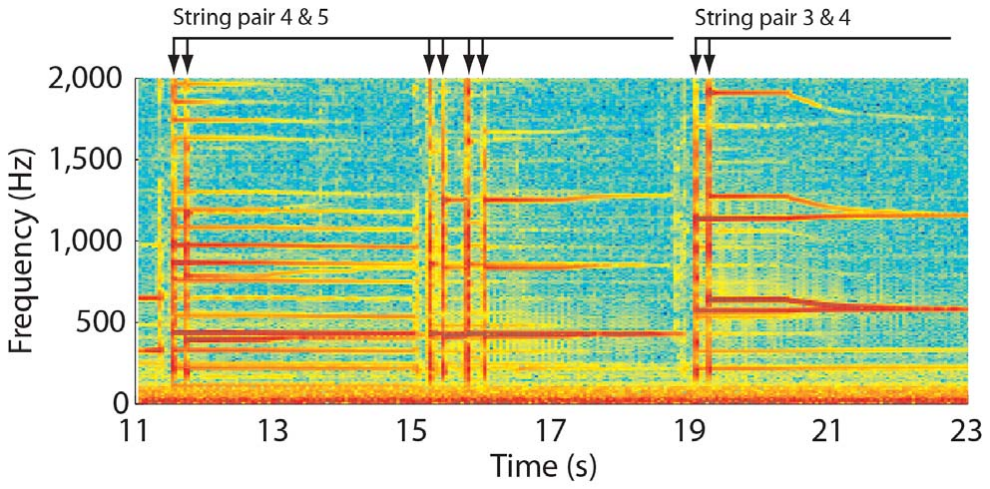
Spectrogram of a guitar being tuned in real time by CI1. Frequency is on the y-axis, and time on the x-axis. Arrows at the top of the plot indicate when the strings were plucked, and the bars delineate each pair of strings.

Figure 1 shows the spectrogram of CI1 tuning his guitar with harmonics, a method that uses higher order vibration patterns to produce tones of the same frequency on adjacent strings (for audio, refer to Audio S1). For example, the 3^rd^ harmonic generated by the 7^th^ fret on the G-string produced the same note as the 4^th^ harmonic on the 5^th^ fret of the D-string (Table S1). Strings 4 & 5 were plucked just before 12 s, and the 4^th^ string was adjusted upwards in pitch towards so that the harmonics from both strings matched in frequency. For pair 3 & 4, the pitch of the 3^rd^ string was adjusted down to match. In about 1 min, CI1 had finished tuning the guitar relative to the 6^th^ string.

**Figure 2:**
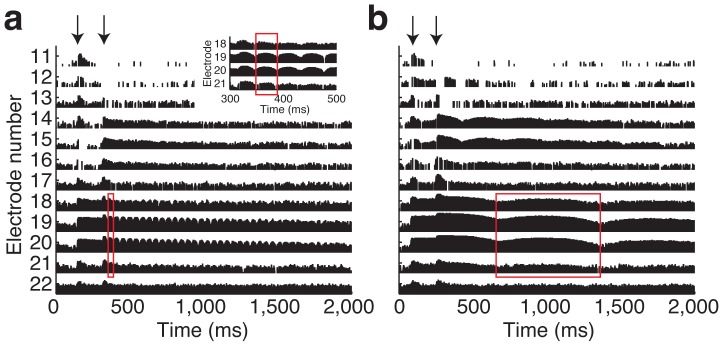
Electrodogram of a CI speech processor output to an out-of-tune and in-tune guitar. Time is represented on the x-axis. Electrode number is on the y-axis. Electrodes 1–11 contained only a small minority of the stimulation pulses and are not shown. The height of each vertical bar in the plot reflects the normalized amplitude of a single stimulation pulse. The red rectangles on each plot highlight a single period of amplitude modulation that is present across multiple electrodes. (**a**) Out-of-tune harmonics from strings 3 and 4. The inset, upper right, shows an expanded view of a short time segment to reveal the details in the amplitude modulation of the stimulation pulses. (**b**) In-tune.

According to CI1, the cue was an audible “vibration” when the frequencies were close but did not match. Commonly known as beats, these amplitude modulations result from the interference pattern of two tones (Fig. S1). Tuning the strings depended on minimizing the rate of beating because the rate is equal to the frequency difference between the two tones. Fig. 2 provides direct evidence for the presence of beats in the electrodograms [24] of the subject's speech processor (RFStats, Hearworks, Pty, Melbourne, Australia). It shows the output of the subject's speech processor in the presence of harmonics (∼588 Hz) played simultaneously on the 3^rd^ and 4^th^ guitar strings. When CI1 considered them out-of-tune (Fig. 2a), electrodes 19 (438–563 Hz) and 20 (563–688 Hz) had a 23 Hz amplitude modulation (AM) of the stimulation pulses. Weaker AM were also evident in neighboring electrodes 18 and 21. These AM are identified by the regularly spaced peaks and valleys in the electrodogram. Longer spacing between peaks and valleys correspond to lower rates of AM. With the strings nearly in tune, AM was ∼1.3 Hz (Fig. 2b).

To eliminate any confounding cues that might come from the complex acoustics of a real guitar and the tactile cues from physical interactions (i.e., vibrations), we tested CI1's ability to match two pure tones under laboratory-controlled conditions (see Methods). With simultaneous tone presentation, CI1's mean error magnitude was 0.3±0.1 Hz (mean±s.d., n = 27). Since level roving produced no statistical difference in this condition (one-way ANOVA, F_2,24_ = 0.043, p = 0.96), the data were pooled. The low error confirms that CI1 was able to tune by beats accurately and reliably and without relying on any tactile cues from the guitar itself.

When beats were eliminated by sequential tone presentation, the accuracy of tuning dropped, particularly with the addition of level roving: Mean errors were 9.4±14.2 Hz (±0 dB rove, n = 10), 25.5±49.2 Hz (±5 dB, n = 9), and 32.7 ±42.2 Hz (±10 dB, n = 10). Although the lower 25^th^ percentile was highly dependent on level roving, the upper 75^th^ percentile was consistently near 30 Hz, likely CI1's true pitch discrimination ability using his CI. At ±10 dB roving, CI1 was able to judge ∼5% difference in frequency without loudness cues, and it would not have been enough to tune his guitar properly.

CINH001 performed similarly with CI, and more interestingly, provided a direct comparison with his normal hearing ear. With simultaneous presentation, CI match error was 0.3±0.2 Hz (n = 9). This was significantly better (unpaired t-test, α = 0.05, p = 0.009) than his normal hearing match error of 0.9±0.6 Hz (n = 10). Presented sequentially, CI match error was 33.1±29.8 Hz (n = 10) while normal hearing match error remained low at 2.0±2.4 Hz (n = 10).

CINH002 showed a similar trend as CINH001 in which tuning with beats produced lower match errors. However, with this non-musician, the accuracy was not as high as with the other two subjects. With simultaneous tones, CI match error was 2.0±2.2 Hz (n = 10) and not significantly different from a normal hearing match error of 5.7±6.5 Hz (n = 10, unpaired t-test, p = 0.154). Presented sequentially, CI match error was 77.3±38.3 Hz (n = 10) while normal hearing match error was 43.8.4±29.7 Hz (n = 10, p = 0.043).

## Discussion

In all three subjects, tuning by beats was accurate and precise despite poor pitch perception through CI as revealed through pitch matching of sequentially presented tones. In the musically trained CI users tested here, pitch discrimination was comparable to those reported for non-musician CI users, which ranged from 0.1 to 1.0 discrimination limens, e.g., 100–1000 Hz for a 1 kHz tone [12]. There was no apparent advantage in pitch perception through their CI as a result of their musical experience. Furthermore, CINH001 and CINH002 showed no benefit to pitch perception with CI despite having normal hearing in their non-implanted ears. This implicates the CI hardware itself and electrode-neural interface as the limiting factor in CI pitch perception as opposed to abnormal neural processing in the central auditory system.

For CINH002, although NH match error was large at ∼44 Hz, her pitch discrimination performance was within the normal range when tested with a 3-interval alternative forced choice paradigm (data not shown). Her discrimination limens at 1000 Hz were 0.02 (16.5±10.6 Hz) for NH and 0.07 (65±43.7 Hz) for CI. The larger match errors reported in this study can be attributed to inexperience with the tuning procedure and a larger tolerance for pitch mismatches. Compared to the more robust psychoacoustic measure of the forced choice paradigm, this study's method-of-adjustment was more susceptible to the participant's subjective assessment in pitch matching.

Despite poor CI pitch perception, both musicians, CI1 and CINH001, reliably matched arbitrary frequencies by listening to beats, effectively bypassing the need for fine pitch discrimination. Many musicians use beat tuning, and CI1 indicated that he had been using this method to tune his guitar before his hearing loss. When the tones were far apart initially, both subjects could rely on spectral place cues, e.g., stimulation on different electrodes, to determine the direction to tune. Once the frequencies were close enough, they could then attend to the beating to make an accurate pitch match. This task required not only hearing the beats, but also quickly judging rate changes to minimize beats.

In listening for beats, CI users are detecting and discriminating amplitude modulations, the same form by which modern speech processors send information to the auditory nerve. Existing data indicate that even non-musician CI users do well at detecting amplitude modulations. Normal hearing listeners are able to detect 5–10% changes in amplitude modulations [25] but CI users can do better at 1–3% [26]. This could explain the difference observed in CINH001 between his CI and normal ear. His superior performance with CI compared to his normal ear suggests that when it comes to tuning with beats, it is possible for CI users to have more accuracy than normal hearing listeners.

**Figure 3:**
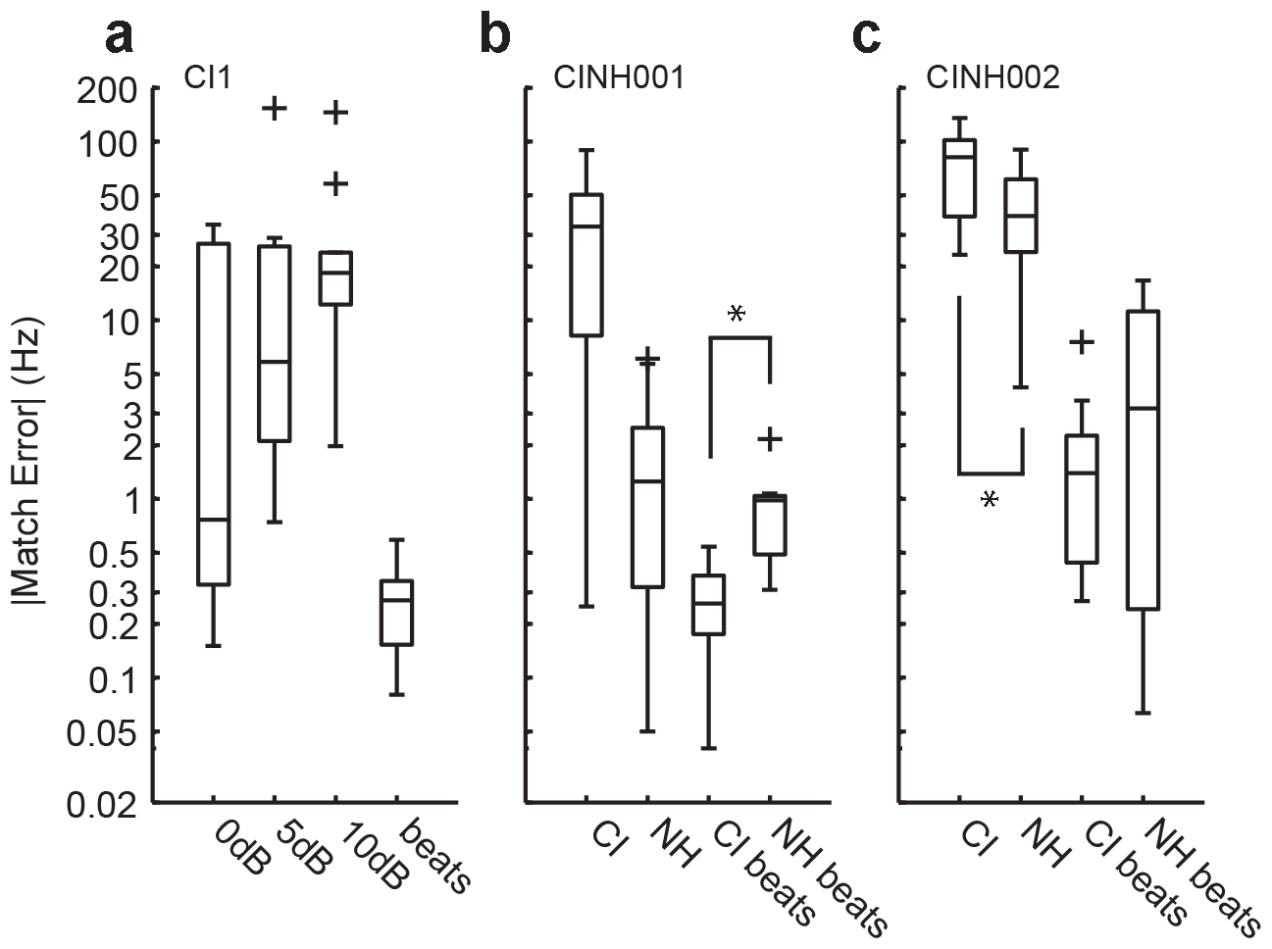
Tuning accuracy. The magnitude of the match error is on the y-axis. The boxplots indicate lower, median, and upper quartile ranges of the data. The whiskers show the full extent of the data. Outliers more than 1.5 s.d. away from are represented by ‘*+*’. (**a**) For CI1, level roving with sequential tone presentation is categorized into *0*, *5* and *10 dB*. Data from simultaneous presentation (*beats*) were pooled since there were no significant differences due to level roving. (**b**) Match errors for CINH001 with his implant (CI) and normal hearing ear (NH). The ‘*’ indicates a statistically significant difference (unpaired t-test, p = 0.009). (**c**) Match errors for CINH002. Format is the same as b. The ‘*’ indicates a statistically significant difference (unpaired t-test, p = 0.043).

Although the CI musicians tested here had above average pitch discrimination of 5–6.5% compared to 10% for the non-musician subject pool (all except one) in Gfeller 2002 [12], they were within the range of variability. Gfeller noted that a few individuals had discrimination of 2–3% which approached NH average of 1%. At least in these two CI musicians, despite strong motivation for good pitch perception, it appears that auditory cortical processing cannot fully recover fine pitch information from CI. Nevertheless, due to his musical experience, it is speculated that CI1 is performing as well as his implant will allow. This is also reflected in CI1's use of loudness cues in pitch matching without level roving (Fig. 3a) and suggests that his overall listening strategy integrates cues related to pitch but which are not explicitly pitch.

Between CI1 and CINH001, their perceptions and musical appreciation through the CI were drastically different. CI1 reported that, initially, many notes on the guitar sounded noisy and indistinguishable and that he spent considerable effort and time before it began sounding musical. In contrast, CINH001 who still had use of his normal ear, maintained that music through the CI was awful. The difference in their reliance on the CI may be partly responsible for their perceived difference in sound quality and also their scores for speech (CINH001: 70% vs. CI1: 99%) and music recognition (7% vs. 55%). The non-musician in this study, CINH002, had the same complaint about CI sound quality and showed similarly low CI scores (speech: 45%; music: 8%). As these differences suggest, maximizing performance with a CI requires extensive training [27], [28]. Even with normal hearing non-musicians, training can improve frequency discrimination performance [29], [30], and in pre-lingually deafened CI children, musical training may help improve pitch perception [31].

Because musical experience in this study's two CI musicians did not appear to confer any advantage for improving pitch perception, it is likely that any neural enhancements for pitch discrimination, as demonstrated by NH musicians, are still not enough to overcome the impoverished spectral content delivered by a CI. Despite generally poor performance in music related tasks, deaf musicians can tune their musical instrument using the CI alone, as demonstrated here. The present report used controlled conditions to not only verify the accurate tuning of a guitar by CI musicians, but also the mechanism underlying this accurate tuning. Instead of using the pitch cue, they used beats to accomplish this seemingly difficult job. Accurate guitar tuning via this spectral to temporal transformation is both surprising and enlightening with respect to CI functionality, demonstrating CI users' resourcefulness in taking advantage of their implants to solve challenging, real-world problems.

## Supporting Information

Figure S1 **Example of beats produced by interference of two different tones.**
(DOCX)

Table S1 **Notes and harmonics on a guitar in standard tuning.**
(DOC)

Audio S1 **Audio demonstration of subject CI1 tuning a guitar in real-time.**
(WAV)
