# Supplementary material for: Accurate Guitar Tuning by Cochlear Implant Musicians
Source: PLoS One. 2014 Mar 20;9(3):e92454. doi: 10.1371/journal.pone.0092454 (PMC3961348; doi:10.1371/journal.pone.0092454)
Supplement: Figure S1 — Example of beats produced by interference of two different tones. (DOCX) [file pone.0092454.s001.docx]

**Figure S1**. Beats are produced by interference of two different tones (upper). Beat rate is equal to the difference in tone frequencies. A 5 Hz amplitude modulation results from 30 Hz and 35 Hz tones (middle). The closer the tones are in frequency, the slower the beat rate. 30 Hz and 32 Hz tones together generate 2 Hz amplitude modulation (lower).
