# Supplementary material for: Accurate Guitar Tuning by Cochlear Implant Musicians
Source: PLoS One. 2014 Mar 20;9(3):e92454. doi: 10.1371/journal.pone.0092454 (PMC3961348; doi:10.1371/journal.pone.0092454)
Supplement: Table S1 — Notes and harmonics on a guitar in standard tuning. (DOC) [file pone.0092454.s002.doc]

## **Table S1.** Notes and harmonics on a guitar in standard tuning.

| **String (Note)** | **Fundamental Frequency** | **3^rd^ harmonic**  **(7^th^ fret)** | **4^th^ harmonic**  **(5^th^ fret)** |
| --- | --- | --- | --- |
| 1 (E4) | 329.6 Hz | 988.9 Hz | 1318.5 Hz |
| 2 (B3) | 246.9 Hz | 740.8 Hz | 987.8 Hz |
| 3 (G3) | 196.0 Hz | 588.0 Hz | 784.0 Hz |
| 4 (D3) | 146.8 Hz | 440.5 Hz | 587.3 Hz |
| 5 (A2) | 110.0 Hz | 330.0 Hz | 440.0 Hz |
| 6 (E2) | 82.4 Hz | 247.2 Hz | 329.6 Hz |
